# Supplementary material for: Ferritin heavy chain supports stability and function of the regulatory T cell lineage
Source: EMBO J. 2024 Mar 18;43(8):4. doi: 10.1038/s44318-024-00064-x (PMC11021483; doi:10.1038/s44318-024-00064-x)
Supplement: Supplementary file 1 — Appendix [file 44318_2024_64_MOESM1_ESM.pdf]

Ferritin heavy chain supports stability and function of the regulatory T cell lineage

Appendix Figures

Table of Contents:

APPENDIX FIGURE S1: *Relative quantification of Fth mRNA.*
APPENDIX FIGURE S2: *Relative quantification of Fth mRNA.*
APPENDIX FIGURE S3: *BLAST analysis of the mouse Fth promoter region.*
APPENDIX FIGURE S4: *Putative FOXP3 binding site in the murine Fth promoter*

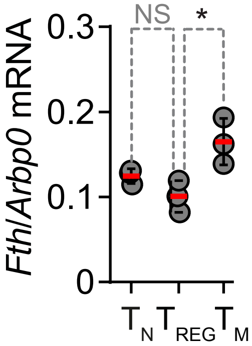

APPENDIX FIGURE S1 LEGEND: Relative quantification of *Fth* mRNA expression in freshly isolated (FACS-sorted from spleen) (CD4$^+$Foxp3$^-$CD44^{low}CD62L^{high}) naïve T cells (T_N), CD4$^+$GFP$^+$ T_{REG} cells and CD4$^+$GFP$^-$CD44^{high}CD62L^{low} memory T cells (T_M) by qRT-PCR, normalized to *Arbp0* mRNA. Data from 3 individual mice, represented as mean ± SD. Circles correspond to individual mice and red bars are mean values. P values calculated using Ordinary one-way ANOVA. NS, not significant (P > 0.05), *P < 0.05. Methods for T_{REG} cell sorting and qRT-PCR are detailed in the manuscript.

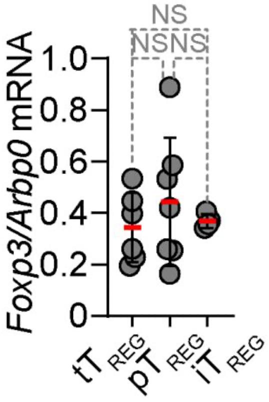

APPENDIX FIGURE S2 LEGEND. Relative quantification of *Fth* mRNA expression in (CD4$^+$GFP$^+$CD25$^+$CD8$^-$B220$^-$CD11b$^-$) T_{REG} cells normalized to *Arbp0*, FACS-sorted from the thymus and lymph nodes as well as in iT_{REG} cells, generated *in vitro* as detailed in *Fig. 1E-G*. Data from N=4-7 mice in four independent experiments. P values calculated with ordinary one-way ANOVA with Tukey's multiple comparisons test and show no significant differences.

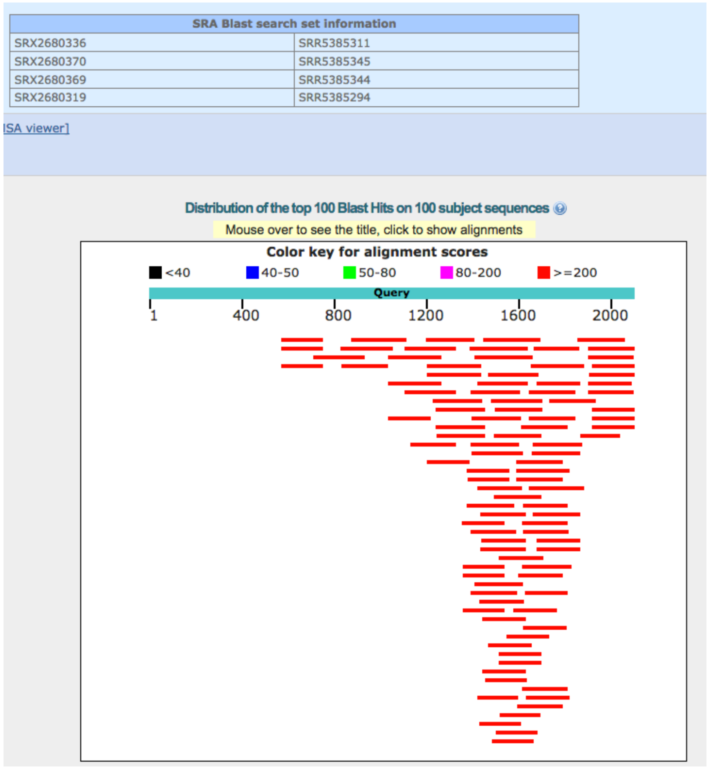

APPENDIX FIGURE S3 LEGEND: Comparison of the mouse Fth promoter region (>FP023586 Fth1_1 :+U EU:NC; range -2000 to 100) with following databases (BLAST analysis):

1. **H3K27ac ChIP-seq of Treg replicate 1:** 1 ION_TORRENT (Ion Torrent Proton) run: 19.6M spots, 2.9G bases, 2.1Gb downloads; Accession: SRX2680370 https://www.ncbi.nlm.nih.gov/sra/SRX2680370[accn]
2. **H3K27ac ChIP-seq of Treg replicate 2;** 1 ION_TORRENT (Ion Torrent Proton) run: 17.7M spots, 2.5G bases, 1.8Gb downloads; Accession: SRX2680369 https://www.ncbi.nlm.nih.gov/sra/SRX2680369[accn]
3. **H3K4me3 ChIP-seq of T$_{REG}$;** 1 ION_TORRENT (Ion Torrent Proton) run: 13.8M spots, 2G bases, 1.4Gb downloads; Accession: SRX2680336; https://www.ncbi.nlm.nih.gov/sra/SRX2680336[accn]
4. **Foxp3 ChIP-seq of T$_{REG}$;** 1 ION_TORRENT (Ion Torrent Proton) run: 30.6M spots, 4.5G bases, 3.2Gb downloads; Accession: SRX2680319; https://www.ncbi.nlm.nih.gov/sra/SRX2680319[accn]

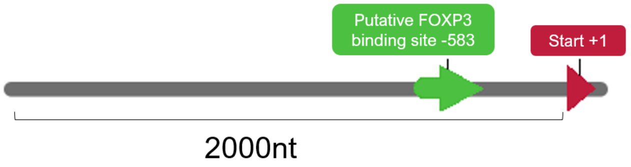

APPENDIX FIGURE S4 LEGEND: Putative FOXP3 binding site in the murine *Fth* promoter analysed using the Eukaryotic Promoter Database (https://epd.expasy.org/epd) with Transcription factor motifs (Jaspar core 2018 vertebrates).
