# Supplementary material for: Ferritin heavy chain supports stability and function of the regulatory T cell lineage
Source: EMBO J. 2024 Mar 18;43(8):4. doi: 10.1038/s44318-024-00064-x (PMC11021483; doi:10.1038/s44318-024-00064-x)

README

FTH and β-Actin protein expression, detected by western blot in whole cell extracts from human T conventional (CD4+CD45RA+CD127+CD25–; TCONV) and TREG (CD4+CD127–CD45RA+CD25hi) cells after two weeks of expansion with anti-CD3, anti-CD28 mAb and IL-2. Two representative experiments are shown

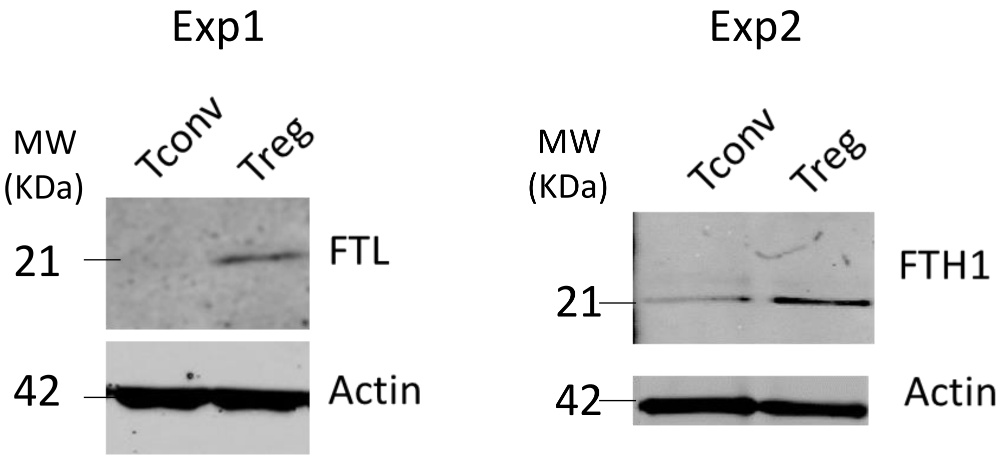

Supplement: Supplementary file 2 — Source Data Fig. 1 [file 44318_2024_64_MOESM2_ESM.zip › Figure_1/1A/figura 1A.pdf]
