# Supplementary figures and images for: Ferritin heavy chain supports stability and function of the regulatory T cell lineage
Source: EMBO J. 2024 Mar 18;43(8):4. doi: 10.1038/s44318-024-00064-x (PMC11021483; doi:10.1038/s44318-024-00064-x)

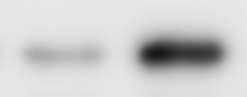

Supplement: Supplementary file 2 — Source Data Fig. 1 [file 44318_2024_64_MOESM2_ESM.zip › Figure_1/1F/Fth.tif]

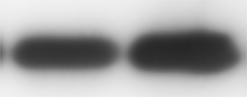

Supplement: Supplementary file 2 — Source Data Fig. 1 [file 44318_2024_64_MOESM2_ESM.zip › Figure_1/1F/h3.tif]

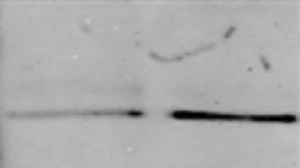

Supplement: Supplementary file 2 — Source Data Fig. 1 [file 44318_2024_64_MOESM2_ESM.zip › Figure_1/1A/Exp2_FTH.tif]

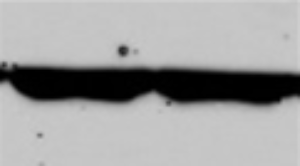

Supplement: Supplementary file 2 — Source Data Fig. 1 [file 44318_2024_64_MOESM2_ESM.zip › Figure_1/1A/Exp1_Actin.tif]

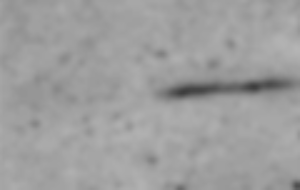

Supplement: Supplementary file 2 — Source Data Fig. 1 [file 44318_2024_64_MOESM2_ESM.zip › Figure_1/1A/Exp1_FTH.tif]

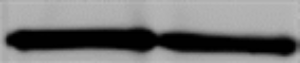

Supplement: Supplementary file 2 — Source Data Fig. 1 [file 44318_2024_64_MOESM2_ESM.zip › Figure_1/1A/Exp2_Actin.tif]

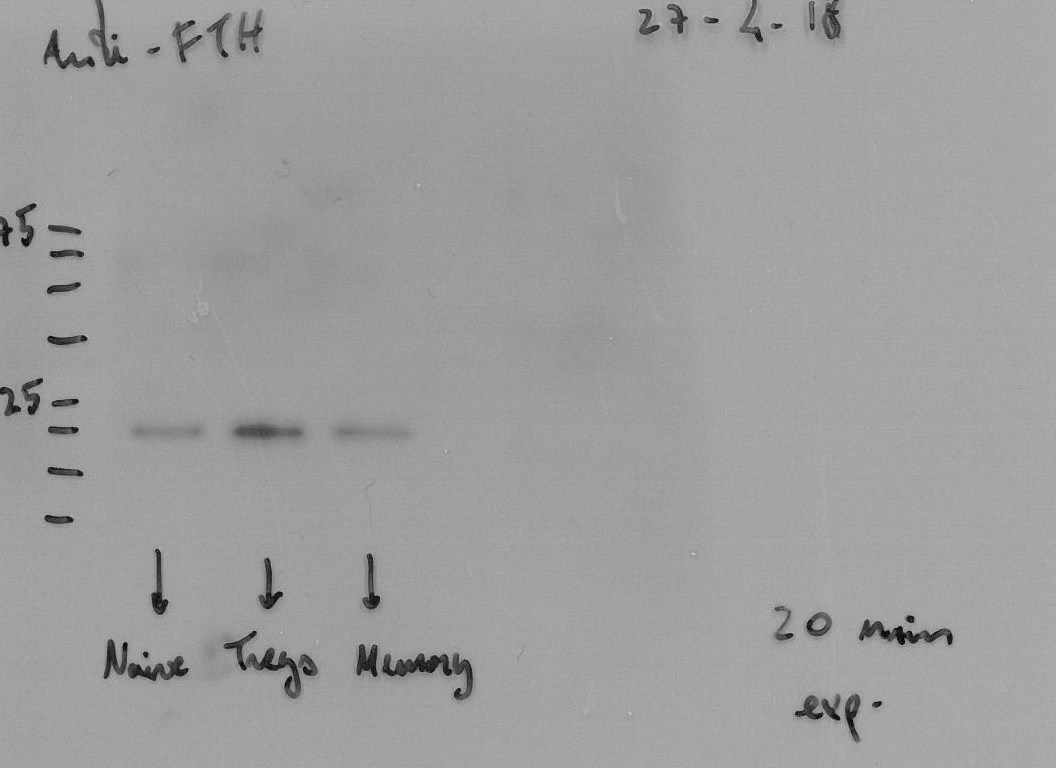

Supplement: Supplementary file 2 — Source Data Fig. 1 [file 44318_2024_64_MOESM2_ESM.zip › Figure_1/1C/fth.tif]

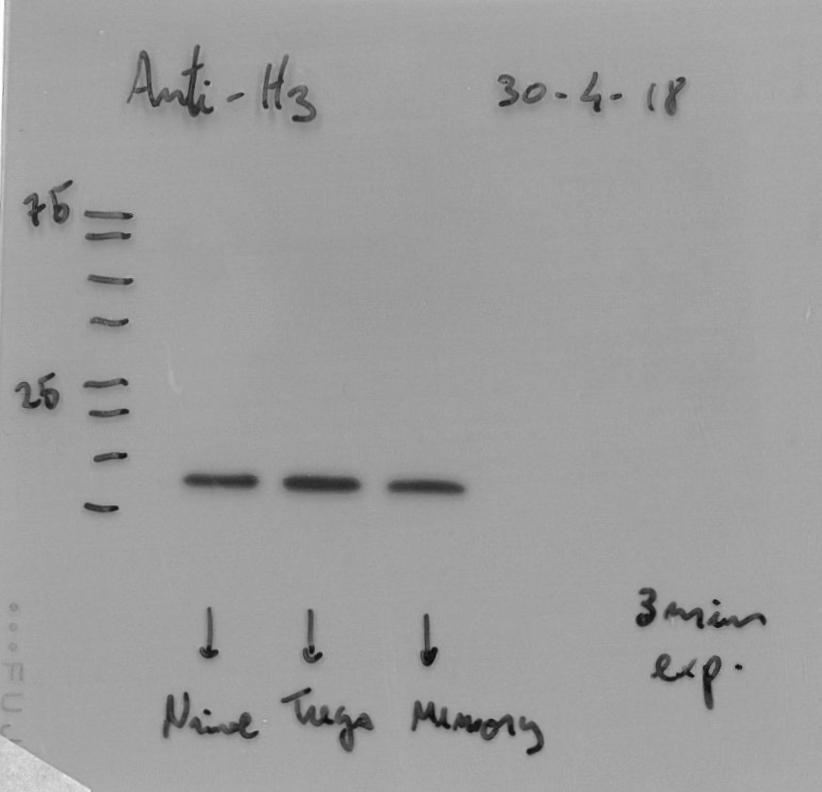

Supplement: Supplementary file 2 — Source Data Fig. 1 [file 44318_2024_64_MOESM2_ESM.zip › Figure_1/1C/h3.tif]

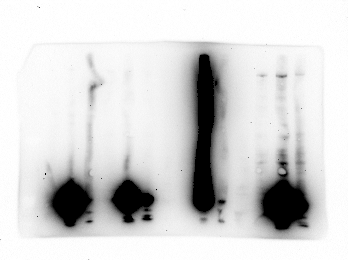

Supplement: Supplementary file 7 — Source Data Fig. 6 [file 44318_2024_64_MOESM7_ESM.zip › Figure_6/6I/TET-flag blot.tif]

95 degree for 10 min  
load 10ug cytoplasm extract and 20ug nuclear protein.

Cytosol

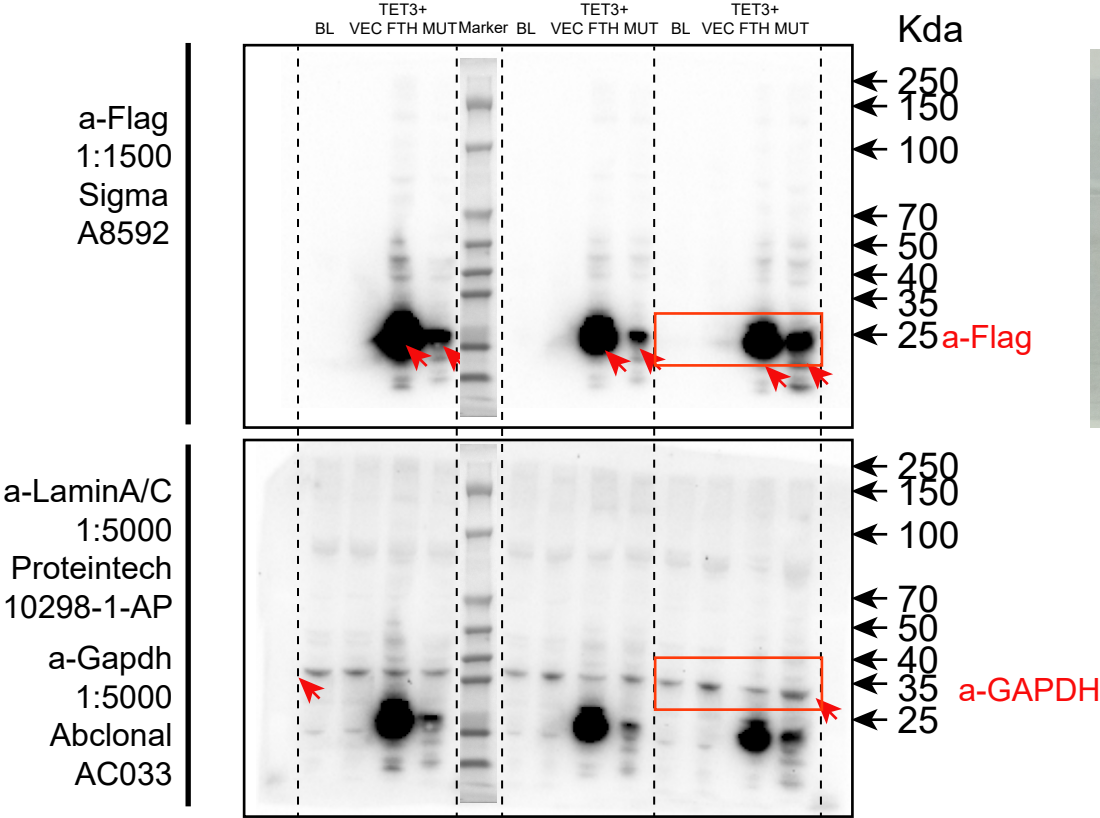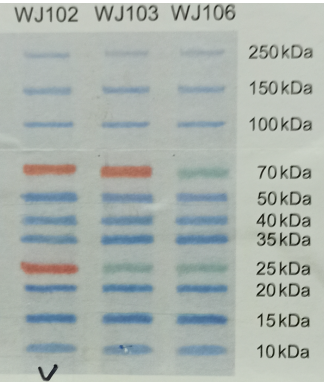

Supplement: Supplementary file 7 — Source Data Fig. 6 [file 44318_2024_64_MOESM7_ESM.zip › Figure_6/6I/Figure 6I right.pdf]

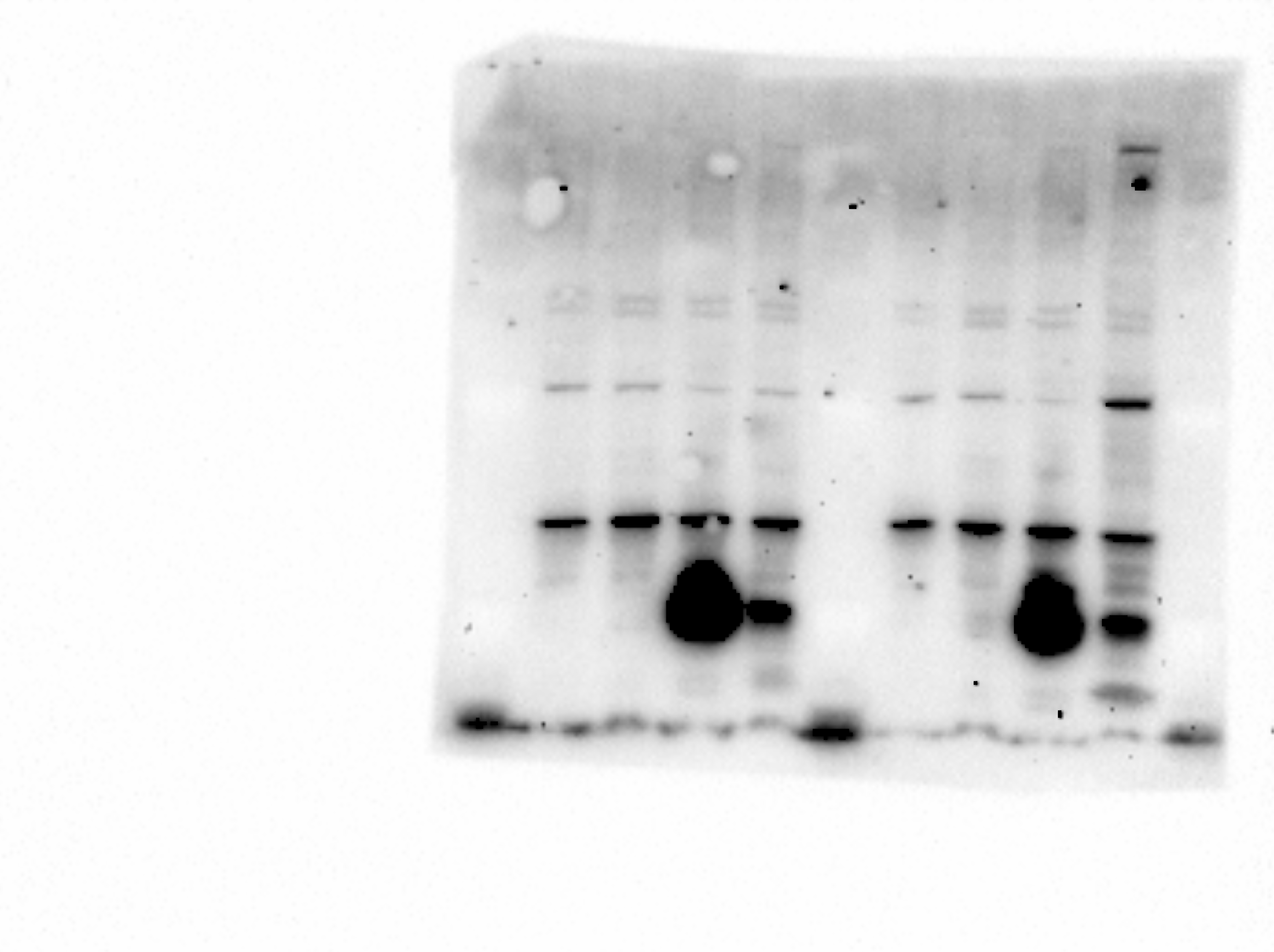

Supplement: Supplementary file 7 — Source Data Fig. 6 [file 44318_2024_64_MOESM7_ESM.zip › Figure_6/6I/gapdh blot.tif]

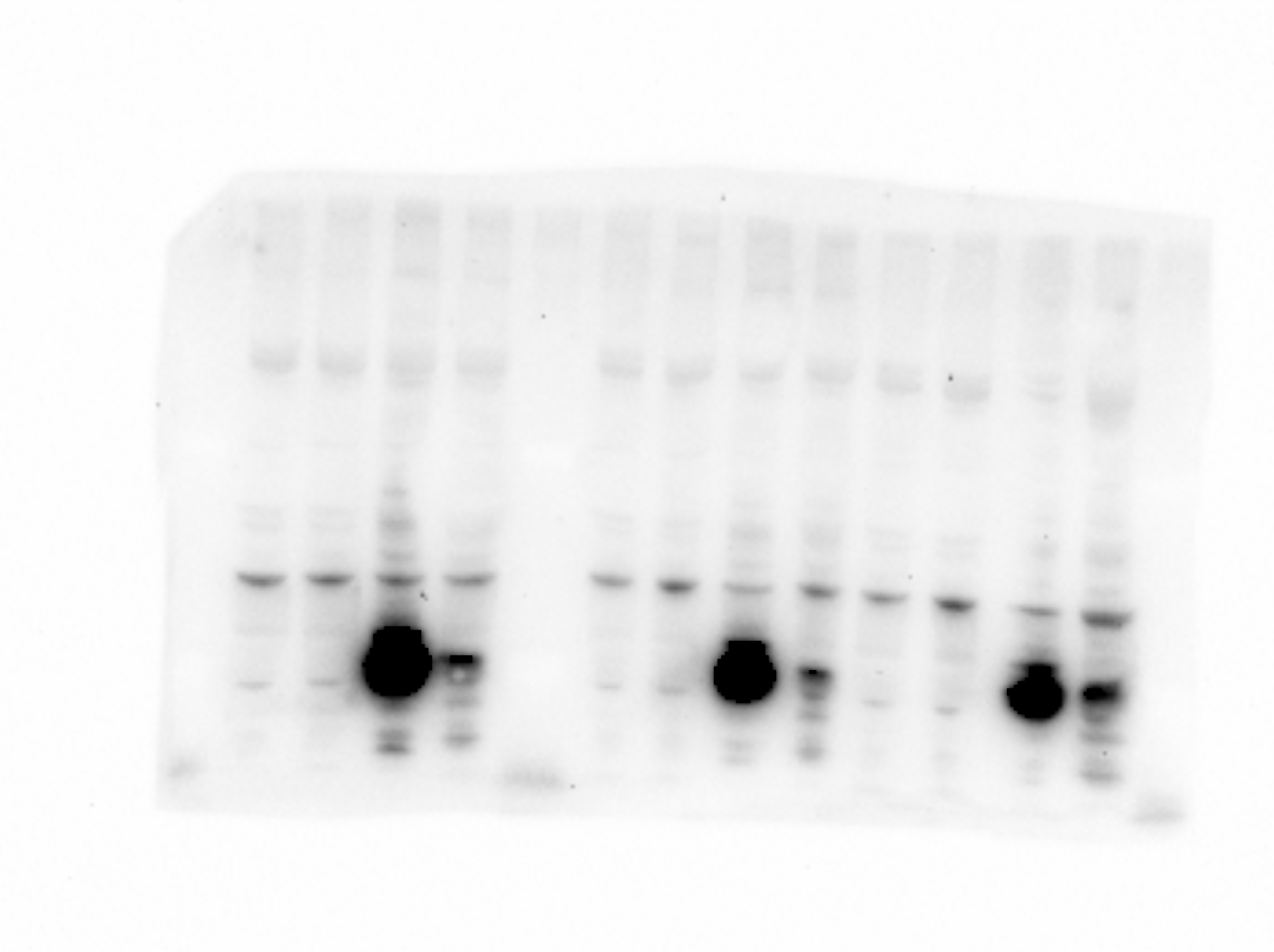

Supplement: Supplementary file 7 — Source Data Fig. 6 [file 44318_2024_64_MOESM7_ESM.zip › Figure_6/6I/FTH-flag blot2.tif]

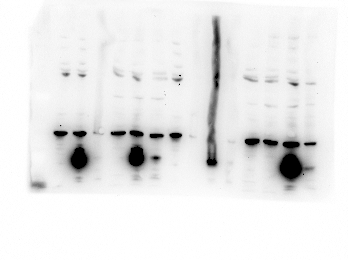

Supplement: Supplementary file 7 — Source Data Fig. 6 [file 44318_2024_64_MOESM7_ESM.zip › Figure_6/6I/lamin blot.tif]

95 degree for 10 min  
load 10ug cytoplasm extract and 20ug nuclear protein.

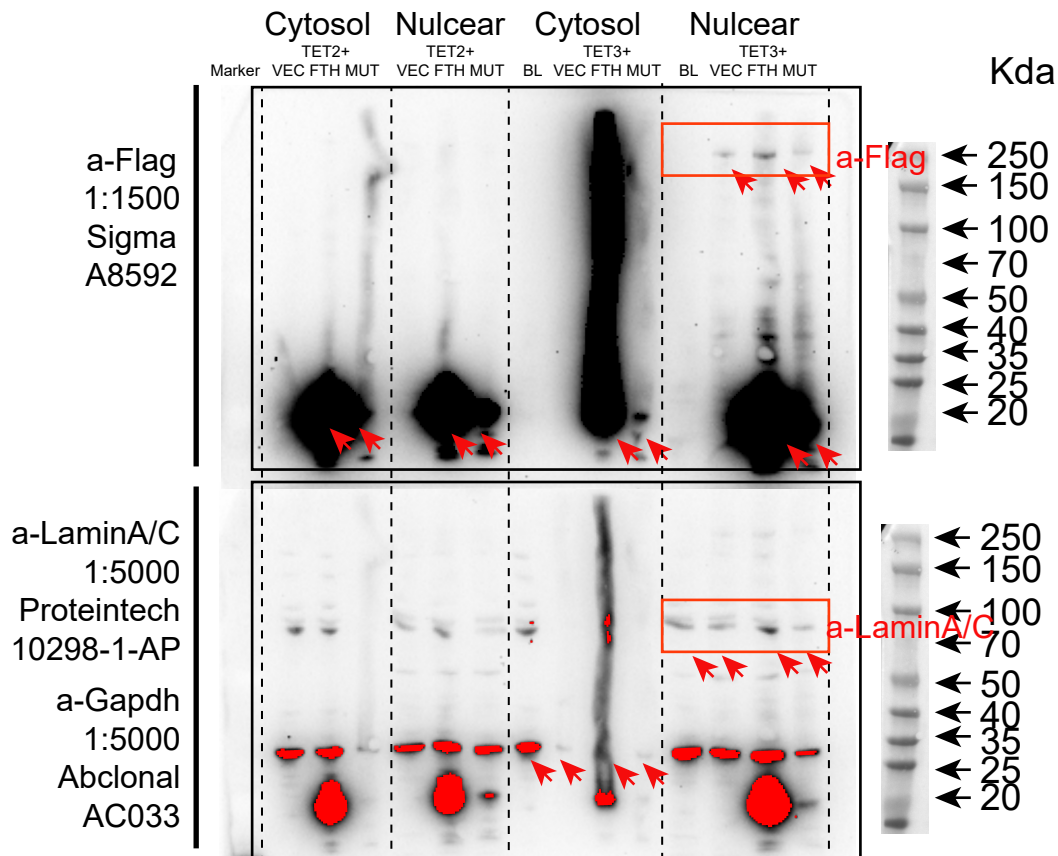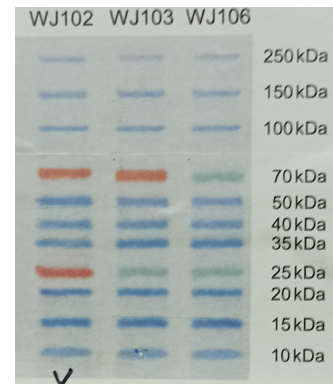

Supplement: Supplementary file 7 — Source Data Fig. 6 [file 44318_2024_64_MOESM7_ESM.zip › Figure_6/6I/Figure6I left.pdf]

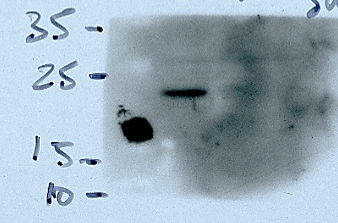

Supplement: Supplementary file 8 — Source Data Fig. 7 [file 44318_2024_64_MOESM8_ESM.zip › Figure_7/7A/FTH.tif]

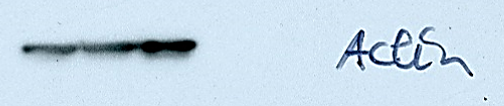

Supplement: Supplementary file 8 — Source Data Fig. 7 [file 44318_2024_64_MOESM8_ESM.zip › Figure_7/7A/Actin.tif]
