# Supplementary material for: Ferritin heavy chain supports stability and function of the regulatory T cell lineage
Source: EMBO J. 2024 Mar 18;43(8):4. doi: 10.1038/s44318-024-00064-x (PMC11021483; doi:10.1038/s44318-024-00064-x)

README

Mean fluorescence intensity (MFI) of FOXP3 expression, detected by flow cytometry in human (CD4+CD45RA+CD25+) TREG cells infected with the recombinant lentiviruses. Representative histogram plot for FOXP3 MFI (left). shControl is shown in blue and shFTH1/2 in dark red. Quantification of Data from N=6 samples per experimental group shown on the right.

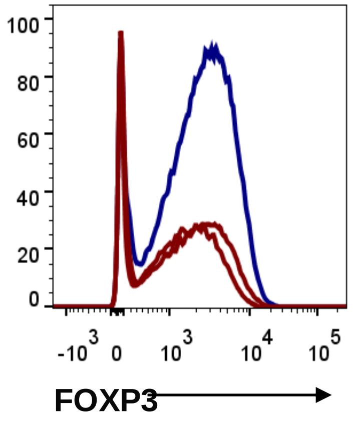

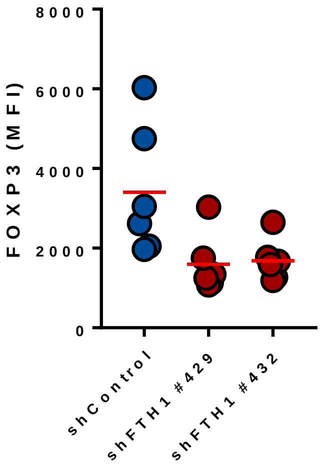

Supplement: Supplementary file 8 — Source Data Fig. 7 [file 44318_2024_64_MOESM8_ESM.zip › Figure_7/7B/Figure_7B.pdf]
