# Supplementary material for: Ferritin heavy chain supports stability and function of the regulatory T cell lineage
Source: EMBO J. 2024 Mar 18;43(8):4. doi: 10.1038/s44318-024-00064-x (PMC11021483; doi:10.1038/s44318-024-00064-x)

README

Representative western blot images of whole cell extracts from HEK293T cells infected with recombinant lentiviruses coding shRNAs targeting FTH (FTH429 and FTH432) or control (Ctrl.) recombinant lentiviruses non-targeting shRNA. Actin was used as loading control.

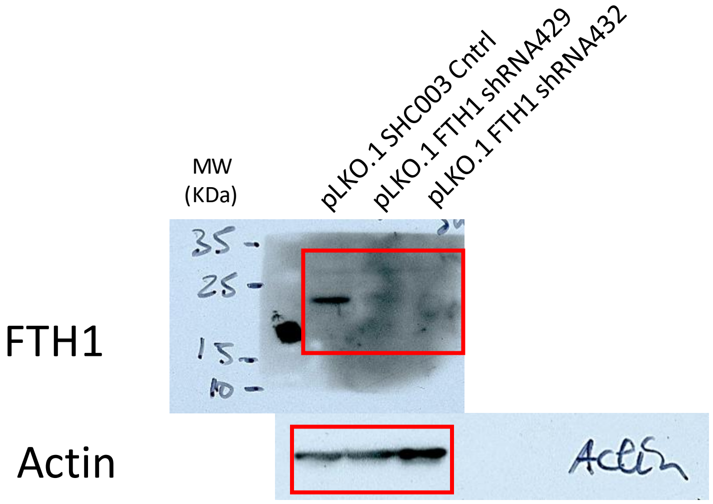

Supplement: Supplementary file 8 — Source Data Fig. 7 [file 44318_2024_64_MOESM8_ESM.zip › Figure_7/7A/Figure_7A.pdf]
