# Supplementary material for: Ferritin heavy chain supports stability and function of the regulatory T cell lineage
Source: EMBO J. 2024 Mar 18;43(8):4. doi: 10.1038/s44318-024-00064-x (PMC11021483; doi:10.1038/s44318-024-00064-x)
Supplement: Supplementary file 10 — EV and Appendix Figure Source Data [file 44318_2024_64_MOESM10_ESM.zip › Source.Files.EV/EV1/1B/1B_DATA.pdf]

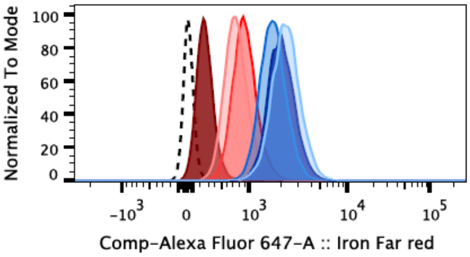

	Sample Name	Subset Name	Count	Geometric Mean : Comp-Alexa Fluor 647-A
	11-7-2019 Vital iron probe_mLN DEL3_012.fcs	Foxp3+	4463	2397
	11-7-2019 Vital iron probe_mLN DEL2_011.fcs	Foxp3+	5615	1811
	11-7-2019 Vital iron probe_mLN DEL1_010.fcs	Foxp3+	3735	2170
	11-7-2019 Vital iron probe_mLN CTR3_009.fcs	Foxp3+	19817	797
	11-7-2019 Vital iron probe_mLN CTR2_008.fcs	Foxp3+	20284	975
	11-7-2019 Vital iron probe_mLN CTR1_007.fcs	Foxp3+	8454	279
	11-7-2019 Vital iron probe_FMO1_013.fcs	Foxp3+	4070	39.9

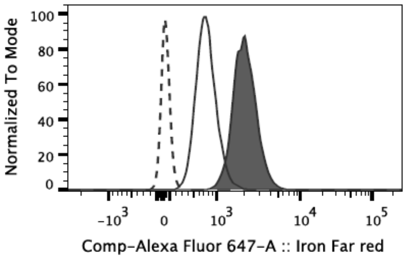

	Sample Name	Subset Name	Count	Geometric Mean : Comp-Alexa Fluor 647-A
	11-7-2019 Vital iron probe_mLN DEL1_010.fcs	Foxp3+	3735	2170
	11-7-2019 Vital iron probe_mLN CTR3_009.fcs	Foxp3+	19817	797
	11-7-2019 Vital iron probe_FMO1_013.fcs	Foxp3+	4070	39.9
