# Supplementary material for: Ferritin heavy chain supports stability and function of the regulatory T cell lineage
Source: EMBO J. 2024 Mar 18;43(8):4. doi: 10.1038/s44318-024-00064-x (PMC11021483; doi:10.1038/s44318-024-00064-x)
Supplement: Supplementary file 12 — Expanded View Figures [file 44318_2024_64_MOESM12_ESM.pdf]

Expanded View Figures

Figure EV1. FTH expression in T_{REG} cells alters systemic iron metabolism.

(**A**) Schematic representation of experimental approach (left panel) and relative quantification of *Fth* mRNA, by qRT-PCR, normalized to *Arbp0* mRNA (right panel), of ($CD4^+$ GFP^+) T_{REG} cells sorted from mesenteric lymph nodes (MLN). Data from $N = 3$ mice per genotype. (**B**) Schematic representation of experimental approach used for relative quantification of intracellular Fe^{2+} in ($CD4^+GFP^+$) T_{REG} cells in mesenteric lymph nodes (MLN), using the FeRhoNox™-1 probe (left panel). Representative flow cytometry histograms (middle panel) and relative quantification (right panel) of mean fluorescence of intracellular Fe^{2+} intensity (MFI) $N = 3$ mice per genotype. (**C**) Schematic representation of experimental approach (left panel), representative flow cytometry dot plots (middle panel) and corresponding quantification of percentage (%) and cell number (Nbr.) (right panels) of live splenic follicular ($CD4^+GFP^+CXCR5^+PD1^+$) FT_{REG} cells. Data from $N = 4$ mice per genotype, from one experiment. (**D**, **E**) Representative flow cytometry dot plots (left panels) and number (right panel) of live activated ($CD4^+CD44^{high}CD62L^{low}$) and ($CD8^+CD44^{high}CD62L^{low}$) T cells in the spleen (**D**) and MLN (**E**). Data from $N = 6$–8 mice per genotype, pooled from four independent experiments, with similar trend. (**F**, **G**) Representative flow cytometry dot plots (left panels) and percentage (%) (right panel) of live activated ($CD3^+CD4^+Foxp3^-IFN\text{-}\gamma^+$; T_H1) T_H1 and ($CD3^+CD8^+Foxp3^-IFN\text{-}\gamma^+$) T_C in the spleen (**F**) and MLN (**G**). Data representative of $N = 5$ mice per genotype, pooled from two independent experiments, with similar trend. Data information: Data in (**A**–**G**) represented as mean ± SD, circles in (**A**, **C**–**G**) correspond to individual mice and red bars to mean values. *P* values in (**A**–**C**) calculated using unpaired *t* test with Welch's correction, and in (**D**–**G**) using two-way ANOVA with Sidak's multiple comparison test. $^*P < 0.05$, $^{**}P < 0.01$, $^{***}P < 0.001$, $^{****}P < 0.0001$. Source data are available online for this figure.

© The Author(s)

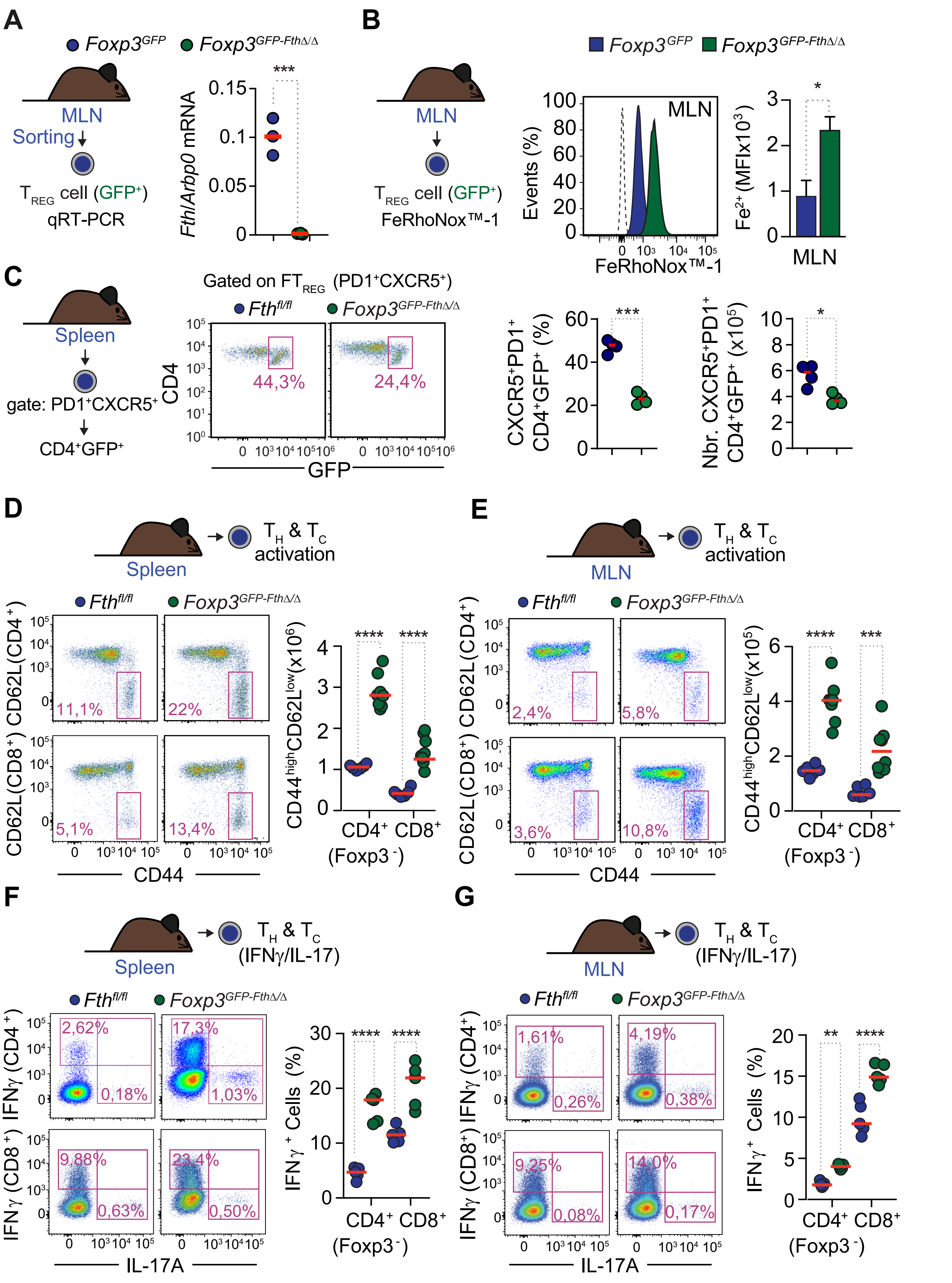
A
Foxp3GFP
Foxp3GFP-FthΔ/Δ
MLN
Sorting
TREG cell (GFP+)
qRT-PCR
Fth/Arbp0 mRNA
***
B
MLN
TREG cell (GFP+)
FeRhoNox™-1
Events (%)
FeRhoNox™-1
Fe2+ (MFIx10³)
*
C
Spleen
gate: PD1+CXCR5+
CD4+GFP+
Gated on FTREG (PD1+CXCR5+)
Fthfl/fl
44,3%
24,4%
CD4
GFP
CXCR5+PD1+ CD4+GFP+ (%)
Nbr. CXCR5+PD1+ CD4+GFP+ (x10⁵)
D
TH & TC activation
11,1%
22%
5,1%
13,4%
CD62L(CD4+)
CD62L(CD8+)
CD44
CD44highCD62Llow(x10⁶)
CD4+ CD8+
(Foxp3-)
****
E
2,4%
5,8%
3,6%
10,8%
CD44highCD62Llow(x10⁵)
F
TH & TC (IFNγ/IL-17)
2,62%
0,18%
17,3%
1,03%
9,88%
0,63%
23,4%
0,50%
IFNγ (CD4+)
IFNγ (CD8+)
IL-17A
IFNγ+ Cells (%)
G
1,61%
0,26%
4,19%
0,38%
9,25%
0,08%
14,0%
0,17%
**

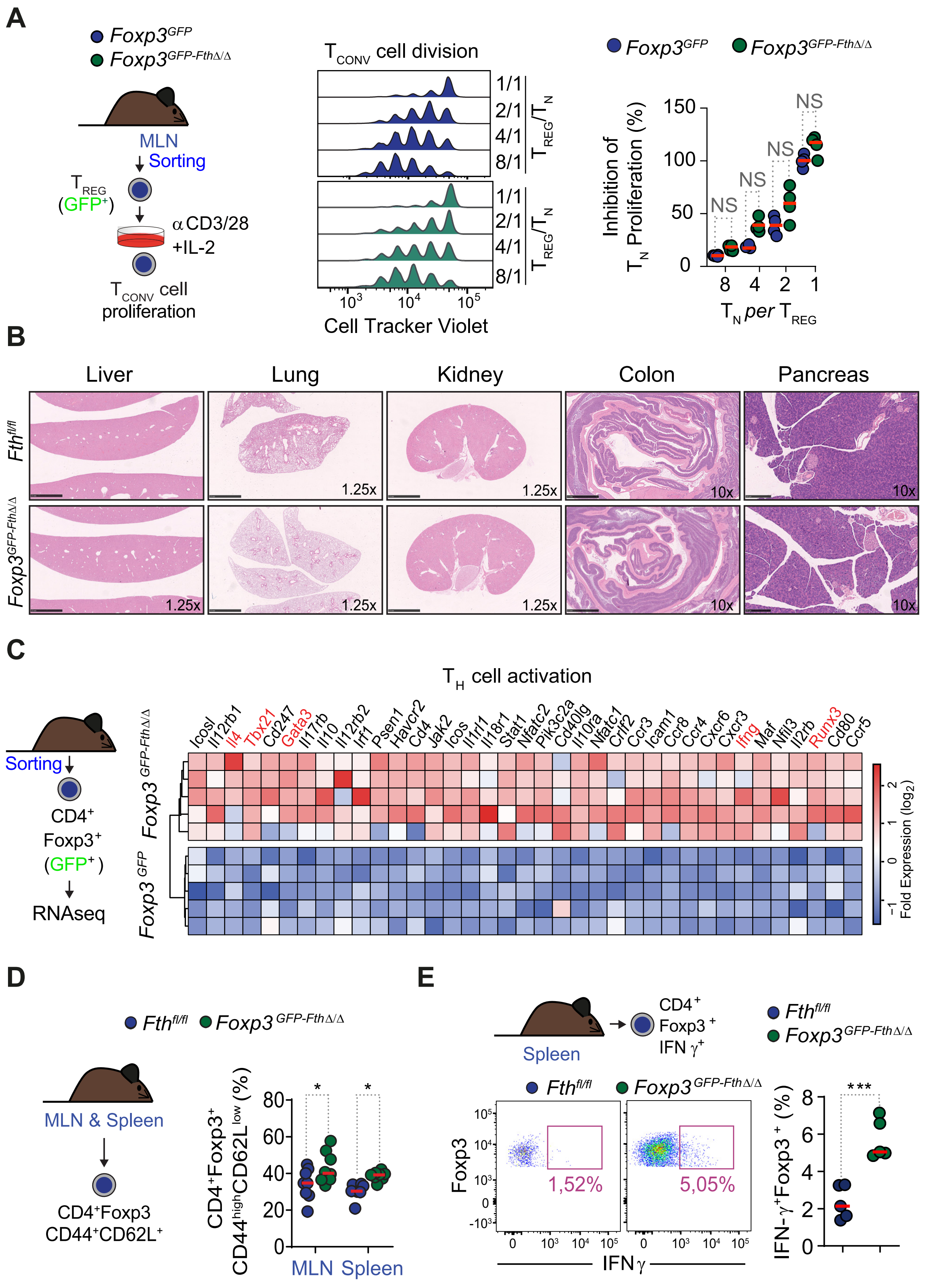
A
Foxp3GFP
Foxp3GFP-FthΔ/Δ
MLN
Sorting
TREG (GFP+)
αCD3/28 +IL-2
TCONV cell proliferation
TCONV cell division
1/1
2/1
4/1
8/1
TREG/TN
Cell Tracker Violet
Inhibition of TN Proliferation (%)
NS
TN per TREG
B
Liver
Lung
Kidney
Colon
Pancreas
Fthfl/fl
Foxp3GFP-FthΔ/Δ
1.25x
10x
C
TH cell activation
Sorting
CD4+ Foxp3+ (GFP+)
RNAseq
Icosl Il12rb1 Il4 Tbx21 Cd247 Gata3 Il17rb Il10 Il12rb2 Irf1 Psen1 Havcr2 Cd4 Jak2 Icos Il1rl1 Il18r1 Stat1 Nfatc2 Pik3c2a Cd40lg Il10ra Nfatc1 Crlf2 Ccr3 Icam1 Ccr8 Ccr4 Cxcr6 Cxcr3 Ifng Maf Nfil3 Il2rb Runx3 Cd80 Ccr5
Fold Expression (log2)
D
Fthfl/fl
Foxp3GFP-FthΔ/Δ
MLN & Spleen
CD4+Foxp3 CD44+CD62L+
CD4+Foxp3+ CD44highCD62Llow (%)
MLN
Spleen
E
Spleen
CD4+ Foxp3+ IFN γ+
Foxp3
IFNγ
1,52%
5,05%
IFN-γ+Foxp3+ (%)

© The Author(s)

Figure EV2. FTH expression in T_{REG} cells prevents systemic cellular inflammation.

(**A**) Schematic representation of experimental approach (left panel) for ($CD4^+CD25^+GFP^+$) T_{REG} cell sorting from the mesenteric lymph nodes (MLN) and coculture with conventional activated T cells (αCD3/28 + IL-2) to evaluate suppressive function of T_{REG} cells. Representative flow cytometry proliferation histograms (Cell Tracer Violet) of in vitro suppression assay of mouse T_N by different ratios of T_{REG} cells (middle panel). Inhibition of T_N cell proliferation quantified as percentage of undivided cells (right panel). Data from 1 out of 3 representative experiments, with similar trend. (**B**) Representative images of H&E-stained liver, lung, kidney, colon, and pancreas from $N = 3$–4 mice per genotype at 27–31 weeks after birth. (**C**) Schematic representation of the experimental approach (left panel) used to generate the Heatmap (right panel) of individual genes associated with T_H effector function programs, differentially expressed in ($CD4^+GFP^+$) T_{REG} cells sorted from *Foxp3*$^{GFP\text{-}Fth\Delta/\Delta}$ vs. *Foxp3*^{GFP} mice (same experiment as Fig. 2A,B). (**D**) Schematic representation of the experimental approach used (left panel) to evaluate the percentage (right panel) of ($CD4^+Foxp3^+CD44^{high}CD62L^{low}$) activated T_{REG} cells in the MLN and spleen. Data from $N = 7$–8 mice per genotype, pooled from three independent experiments, with similar trend. (**E**) Schematic representation of the experimental approach used (top panel), representative flow cytometry dot plots (bottom left panel) and percentage (bottom right panel) of splenic ($CD4^+Foxp3^+$) IFNγ-secreting T_{REG}. Data from $N = 5$ mice per genotype, pooled from two independent experiments, with similar trend. Data information: Data in (**A**, **D**, **E**) represented as mean ± SD. Circles in (**A**) represent individual wells, and red bars are mean values. Circles in (**D**, **E**) represent individual mice, and red bars are mean values. *P* values in (**A**, **D**) calculated using Two-way ANOVA with Sidak's multiple comparison test and in (**E**) using unpaired *t* test with Welch's correction. NS not significant ($P > 0.05$), $^*P < 0.05$; $^{***}P < 0.001$. Source data are available online for this figure.

© The Author(s)

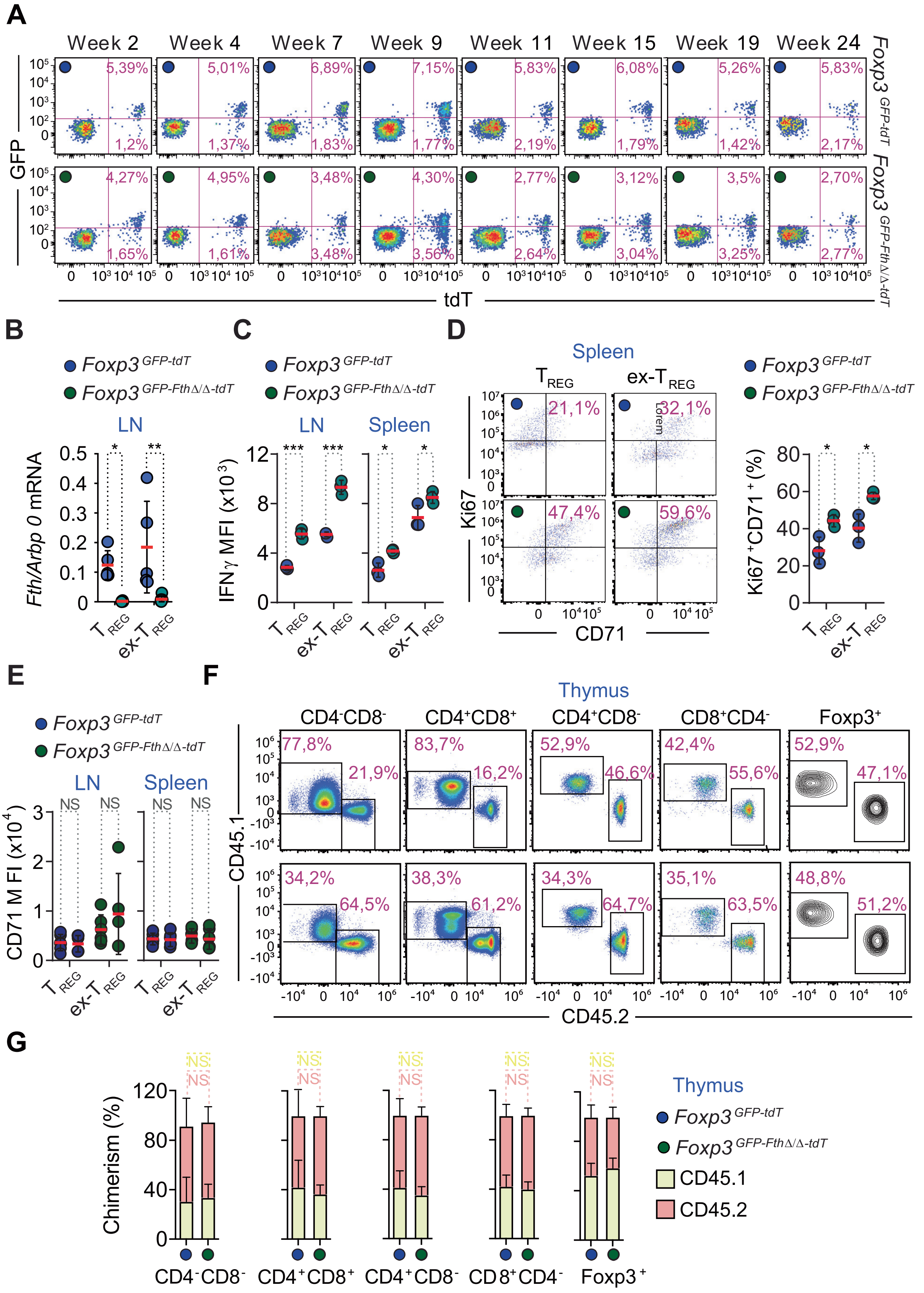
A
Week 2
Week 4
Week 7
Week 9
Week 11
Week 15
Week 19
Week 24
Foxp3 GFP-tdT
Foxp3 GFP-FthΔ/Δ-tdT
GFP
tdT
B
LN
Fth/Arbp 0 mRNA
C
LN
Spleen
IFNγ MFI (x10³)
D
Spleen
Ki67
CD71
Ki67⁺CD71⁺ (%)
E
LN
Spleen
CD71 MFI (x10⁴)
F
Thymus
CD4⁻CD8⁻
CD4⁺CD8⁺
CD4⁺CD8⁻
CD8⁺CD4⁻
Foxp3⁺
CD45.1
CD45.2
G
Thymus
Chimerism (%)
CD45.1
CD45.2

 © The Author(s)

Figure EV3. FTH expression in T_{REG} cells prevents T_{REG} transdifferentiation into inflammatory T_{REG} cells.

(**A**) Representative flow cytometry dot plots of GFP and tdT expression in circulating $CD4^+$ cells (same experiment as Fig. 3A–D). Numbers in quadrants correspond to percentages of positive cells at the indicated weeks after birth. (**B**) Relative quantification of *Fth* mRNA, by qRT-PCR, normalized to *Arbp0* mRNA, in ($CD4^+GFP^+tdT^+$) T_{REG} and ($CD4^+GFP^-tdT^+$) ex-T_{REG} cells sorted from the lymph nodes (LN). Data from $N = 5$–7 mice per genotype, pooled from two experiments with similar trend. (**C**) Mean fluorescence intensity (MFI) of IFNγ in activated ($CD4^+GFP^+tdT^+$) T_{REG} cells and ($CD4^+GFP^-tdT^+$) ex-T_{REG} from the lymph nodes (LN) and spleen in the same experiment as (Fig. 3H). Data from $N = 3$ wells per genotype in one experiment, representative of 2 independent experiments with similar trend. (**D**) Representative flow cytometry dot plots (left panel) and corresponding percentage of $Ki67^+CD71^+$ (right panel) among splenic ($CD4^+GFP^+TdT^+$) T_{REG} cells and ($CD4^+GFP^-TdT^+$) ex-T_{REG} cells. Data from $N = 6$ mice per genotype, pooled from two independent experiments, with similar trend. (**E**) Mean fluorescence intensity (MFI) of CD71 expression in $Ki67^+$ T_{REG} and ex-T_{REG} cells from the lymph nodes (LN) and spleen, from the same experiments as in (**D**). (**F**) Representative flow cytometry dot plots and (**G**) corresponding percentages of $CD45.1^+$ and $CD45.2^+$ double negative (DN), double positive (DP) thymocytes, T_H cells, cytotoxic T cells and ($CD4^+Foxp3^+$) T_{REG} cells in the thymus from the same BM chimeric mice illustrated in (Fig. 4A–G). Data from $N = 11$–12 mice *per* genotype, pooled from two independent experiments with similar trend. Data information: Data in (**B**–**E**, **G**) are presented as mean ± SD, circles in (**B**, **D**, **E**) correspond to individual mice or individual wells (**C**) and red bars are mean values. *P* values in Panel (**B**–**E**, **G**) were calculated using two-way ANOVA with Sidak's multiple comparison test. NS not significant ($P > 0.05$), $*P < 0.05$; $**P < 0.01$; $***P < 0.001$. Source data are available online for this figure.

© The Author(s)

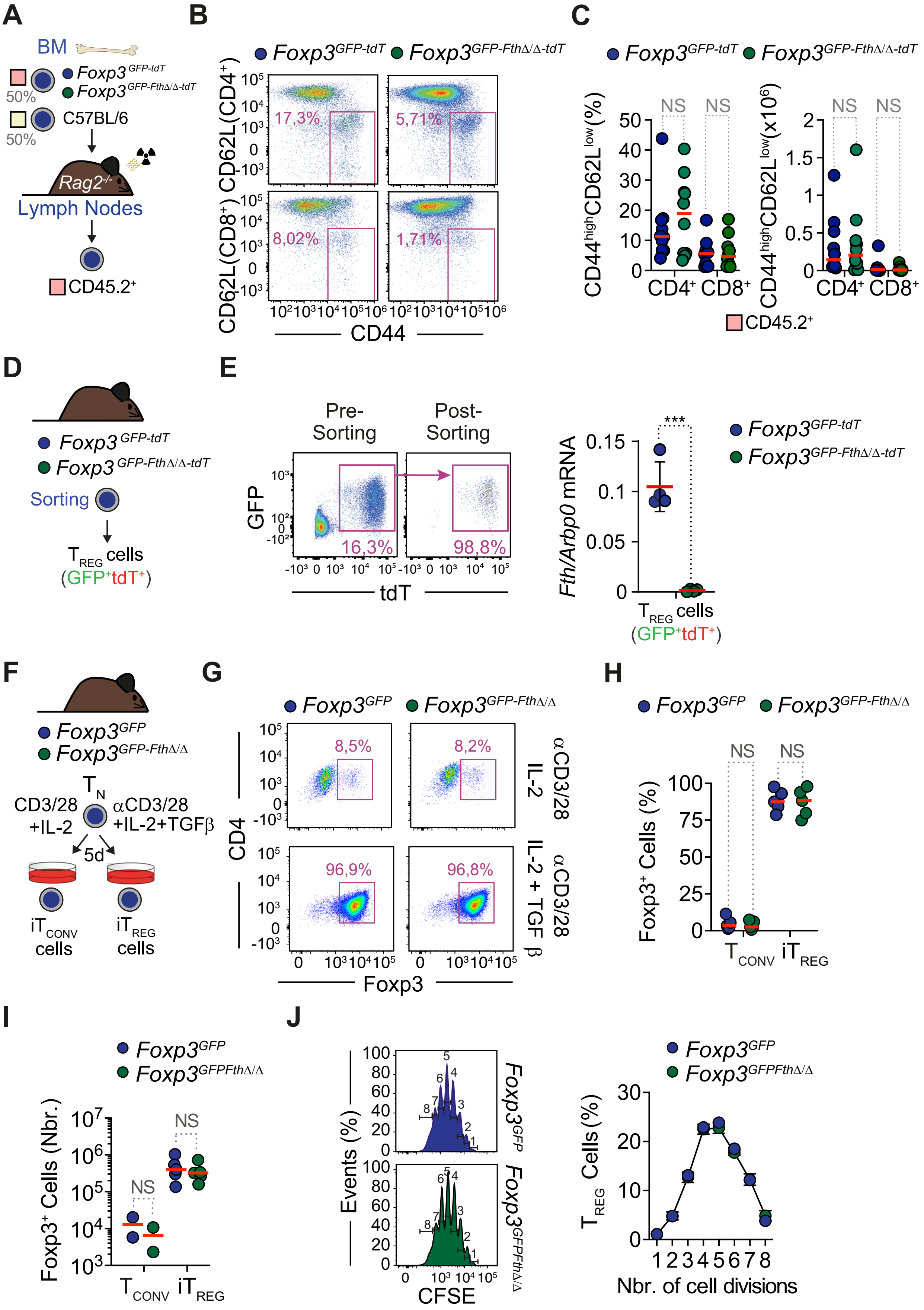
A
BM
Foxp3GFP-tdT
Foxp3GFP-FthΔ/Δ-tdT
50%
C57BL/6
50%
Rag2-/-
Lymph Nodes
CD45.2+
B
Foxp3GFP-tdT
Foxp3GFP-FthΔ/Δ-tdT
CD62L(CD4+)
CD62L(CD8+)
17,3%
5,71%
8,02%
1,71%
CD44
C
Foxp3GFP-tdT
Foxp3GFP-FthΔ/Δ-tdT
NS
CD44highCD62Llow (%)
CD44highCD62Llow (x10^6)
CD4+ CD8+
CD45.2+
D
Foxp3GFP-tdT
Foxp3GFP-FthΔ/Δ-tdT
Sorting
TREG cells
(GFP+tdT+)
E
Pre-Sorting
Post-Sorting
GFP
16,3%
98,8%
tdT
***
Fth/Arbp0 mRNA
TREG cells
(GFP+tdT+)
Foxp3GFP-tdT
Foxp3GFP-FthΔ/Δ-tdT
F
Foxp3GFP
Foxp3GFP-FthΔ/Δ
TN
CD3/28
+IL-2
αCD3/28
+IL-2+TGFβ
5d
iTCONV cells
iTREG cells
G
Foxp3GFP
Foxp3GFP-FthΔ/Δ
8,5%
8,2%
96,9%
96,8%
αCD3/28
IL-2
αCD3/28
IL-2 + TGF β
CD4
Foxp3
H
Foxp3GFP
Foxp3GFP-FthΔ/Δ
NS
Foxp3+ Cells (%)
TCONV
iTREG
I
Foxp3GFP
Foxp3GFPFthΔ/Δ
NS
Foxp3+ Cells (Nbr.)
TCONV
iTREG
J
Foxp3GFP
Foxp3GFPFthΔ/Δ
Events (%)
CFSE
Foxp3GFP
Foxp3GFPFthΔ/Δ
TREG Cells (%)
Nbr. of cell divisions

© The Author(s)

Figure EV4. FTH acts in a non-cell-autonomous manner to prevent T_{REG} cells from transdifferentiating into inflammatory T_{REG} cells.

(**A**) Schematic representation of the experimental approach used for flow cytometry analysis from the lymph nodes of BM chimeric mice (same experiment as Fig. 4A). (**B**, **C**) Representative flow cytometry dot plots (**B**), quantification of percentage (left panel) and number (right panel) (**C**) of live activated ($CD45.2^+CD4^+CD44^{high}CD62L^{low}$) and ($CD45.2^+CD8^+CD44^{high}CD62L^{low}$) cells in the lymph nodes of BM chimeric mice from (**A**). Data in (**C**) from $n = 11–12$ mice per genotype, pooled from 2 independent experiments, with similar trend. (**D**) Schematic representation of cell sorting for adoptive transfers in the experiment illustrated in Fig. 5A. (**E**) Representative flow cytometry dot plots of ($CD4^+GFP^+tdT^+$) T_{REG} cells and relative level of *Fth* mRNA expression in ($CD4^+GFP^+tdT^+$) T_{REG} cells (right panel) used for adoptive transfers in the experiment illustrated in Fig. 5A. (**F**) Schematic representation of experimental approach used for in vitro generation of induced T_{REG} (iT_{REG}) cells and conventional T_H (T_{CONV}) cells from sorted naive T_H (T_N) cells, activated with anti-CD3 and anti-CD28 mAb plus IL-2 and TGFβ. (**G**) Representative flow cytometry dot plots of iT_{REG} and T_{CONV} cells, generated in (**F**). (**H**) Percentage (%) and (**I**) Number (Nbr.) of $Foxp3^+$ T_{CONV} and iT_{REG} cells, generated as described in (**F**). $N = 2–5$ independent experiments with similar trend. Each experiment corresponds to the average of different wells. (**J**) Representative flow cytometry carboxyfluorescein succinimidyl ester (CFSE) staining (left panel) and quantification of percentage (%) (right panel) of proliferating ($CD4^+Foxp3^+$) iT_{REG} cells, generated as described in (**F**). Data from 3 to 6 technical replicates in 1 out of 3 independent experiments, with similar trend. Data information: Data in (**C**, **E**) are presented as mean ± SD, circles correspond to individual mice and red bars are mean values. Circles in (**H–J**) correspond to individual wells and red bars are mean values. *P* values in panels (**C**, **H**, **I**) were calculated using two-way ANOVA with Sidak's multiple comparison test. *P* values in (**E**) were calculated using Mann–Whitney test. NS not significant, $^{***}P < 0.001$. Source data are available online for this figure.

© The Author(s)

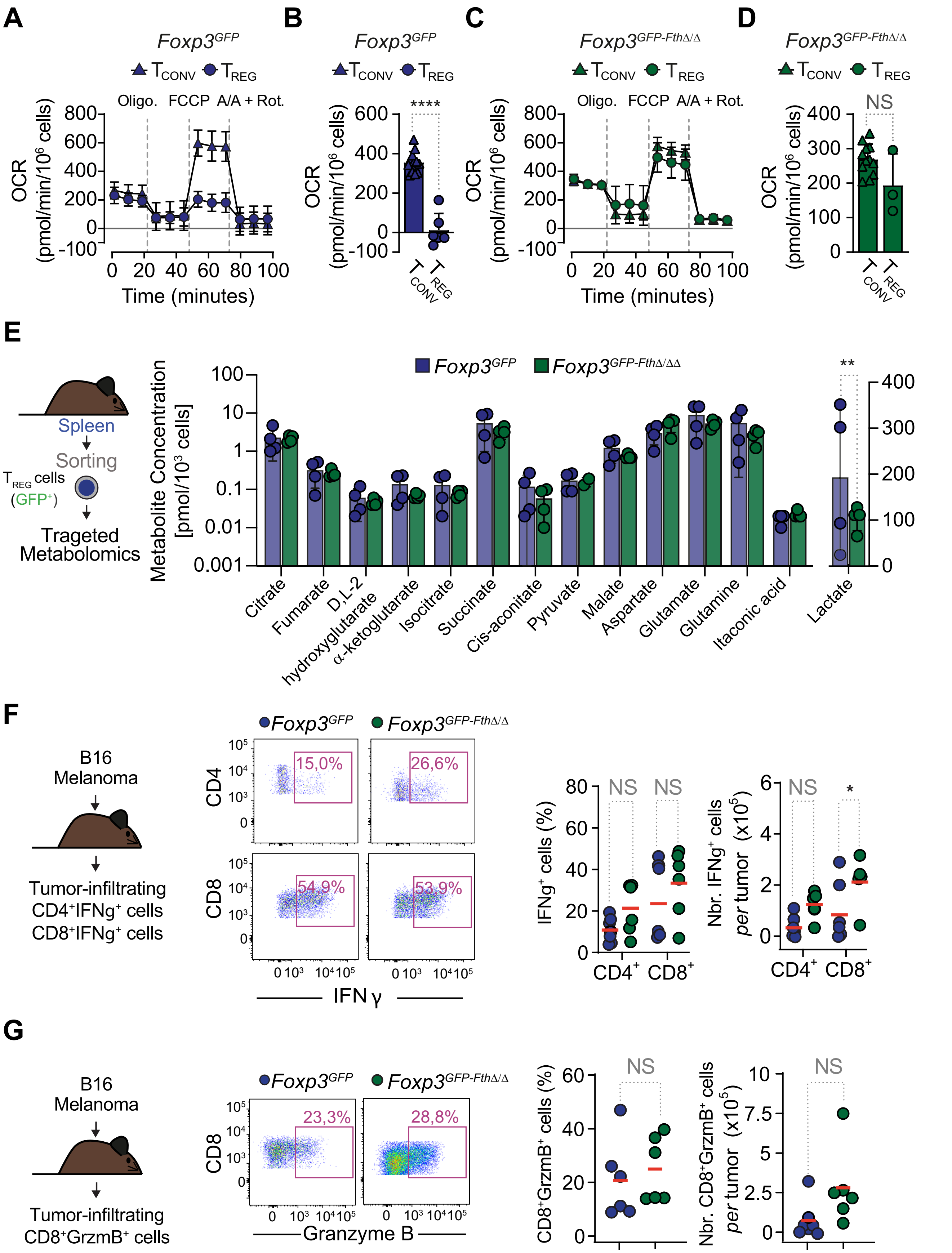
A
Foxp3GFP
TCONV TREG
Oligo. FCCP A/A + Rot.
OCR (pmol/min/10⁶ cells)
Time (minutes)
B
Foxp3GFP
TCONV TREG
****
OCR (pmol/min/10⁶ cells)
C
Foxp3GFP-FthΔ/Δ
TCONV TREG
Oligo. FCCP A/A + Rot.
OCR (pmol/min/10⁶ cells)
Time (minutes)
D
Foxp3GFP-FthΔ/Δ
TCONV TREG
NS
OCR (pmol/min/10⁶ cells)
E
Spleen
Sorting
TREG cells (GFP+)
Trageted Metabolomics
Foxp3GFP Foxp3GFP-FthΔ/ΔΔ
Metabolite Concentration [pmol/10³ cells]
**
Citrate
Fumarate
D,L-2 hydroxyglutarate
α-ketoglutarate
Isocitrate
Succinate
Cis-aconitate
Pyruvate
Malate
Aspartate
Glutamate
Glutamine
Itaconic acid
Lactate
F
B16 Melanoma
Tumor-infiltrating CD4+IFNg+ cells CD8+IFNg+ cells
Foxp3GFP Foxp3GFP-FthΔ/Δ
15,0%
26,6%
54,9%
53,9%
CD4
CD8
IFN γ
NS NS
IFNg+ cells (%)
NS *
Nbr. IFNg+ cells per tumor (x10⁵)
CD4+ CD8+
G
B16 Melanoma
Tumor-infiltrating CD8+GrzmB+ cells
Foxp3GFP Foxp3GFP-FthΔ/Δ
23,3%
28,8%
CD8
Granzyme B
NS
CD8+GrzmB+ cells (%)
NS
Nbr. CD8+GrzmB+ cells per tumor (x10⁵)

 © The Author(s)

Figure EV5. FTH regulates mitochondrial energy metabolism and CPG methylation in T_{REG} cells.

(**A**) Oxygen consumption rate (OCR) in live splenic ($CD4^+GFP^+$) T_{REG} cells and ($CD4^+GFP^-$) T_{CONV} cells from *Foxp3*^{GFP} mice. (**B**) Quantification of spare respiratory capacity, from data represented in (**A**). (**C**) Oxygen consumption rate (OCR) in live splenic ($CD4^+GFP^+$) T_{REG} cells and ($CD4^+GFP^-$) T_{CONV} cells from *Foxp3*$^{GFP\text{-}Fth\Delta/\Delta}$ mice. (**D**) Quantification of spare respiratory capacity, from data represented in (**C**). Data in (**A–D**) pooled from *N* = 3 mice per genotype, represented as mean ± SD. *N* = 3–5 technical replicates in 1 out of 3 independent experiments, with similar trend. Oligomycin (Oligo.), carbonilcyanide p-triflouromethoxyphenylhydrazone (FCCP), Antimycin A/Rotenone (A/A+Rot.). (**E**) Schematic representation of sorting of splenic ($CD4^+GFP^+$) T_{REG} cells used for targeted metabolomics (left panel). Quantification of intermediate metabolites from targeted metabolomics analyzes of splenic T_{REG} cells (right panel). Data from *N* = 3–4 mice per genotype in one experiment representative of 3 independent experiments with similar trend. (**F**) Schematic representation of the experimental approach used for flow cytometry analysis of tumor-infiltrating cells (left panel), representative flow cytometry dot plots (middle panel) and corresponding percentage and number (right panel) of live tumor-infiltrating ($CD4^+IFN\gamma^+$) T_H cells ($CD8^+IFN\gamma^+$) T_C cells, 3 weeks after tumor inoculation (2×10^5 B16 cells). Data from *N* = 6 mice per genotype, pooled from two independent experiments, with similar trend. (**G**) Schematic representation of the experimental approach used for flow cytometry analysis of tumor-infiltrating cells (left panel), representative flow cytometry dot plots (middle panel) and corresponding percentage and number (right panels) of live tumor-infiltrating ($CD8^+GrzmB^+$) T cells, 3 weeks after tumor inoculation (2×10^5 B16 cells). Data from *N* = 6 mice per genotype, pooled from two independent experiments, with similar trend. Data information: Circles and triangles in (**A**, **C**) correspond to mean values, circles, and triangles in (**B**, **D**) correspond to individual wells, and circles in (**E–G**) correspond to individual mice, and red bars are mean values. *P* values in (**A**, **C**) calculated using two-way ANOVA with Bonferroni's (**A**, **C**) or Sidak´s (**E**, **F**) multiple comparisons test, in (**B**, **D**, **G**) using unpaired *t* test with Welch's correction. NS, not significant ($P > 0.05$); $^*P < 0.05$; $^{**}P < 0.01$; $^{****}P < 0.0001$. Source data are available online for this figure.

© The Author(s)
